# Dissolved Organic Carbon Source Influences Tropical Coastal Heterotrophic Bacterioplankton Response to Experimental Warming

**DOI:** 10.3389/fmicb.2019.02807

**Published:** 2019-12-05

**Authors:** Christian Lønborg, Federico Baltar, Cátia Carreira, Xosé Anxelu G. Morán

**Affiliations:** ^1^Australian Institute of Marine Science, Townsville, QLD, Australia; ^2^Department of Limnology and Bio-Oceanography, University of Vienna, Vienna, Austria; ^3^Department of Marine Science, University of Otago, Dunedin, New Zealand; ^4^Departamento de Biologia and CESAM, Universidade de Aveiro, Aveiro, Portugal; ^5^Red Sea Research Center, King Abdullah University of Science and Technology, Thuwal, Saudi Arabia

**Keywords:** dissolved organic carbon, temperature, microbial carbon cycling, extracellular enzymatic activity, tropical coastal waters, Great Barrier Reef

## Abstract

Global change impacts on marine biogeochemistry will be partly mediated by heterotrophic bacteria. Besides ocean warming, future environmental changes have been suggested to affect the quantity and quality of organic matter available for bacterial growth. However, it is yet to be determined in what way warming and changing substrate conditions will impact marine heterotrophic bacteria activity. Using short-term (4 days) experiments conducted at three temperatures (−3°C, *in situ*, +3°C) we assessed the temperature dependence of bacterial cycling of marine surface water used as a control and three different dissolved organic carbon (DOC) substrates (glucose, seagrass, and mangrove) in tropical coastal waters of the Great Barrier Reef, Australia. Our study shows that DOC source had the largest effect on the measured bacterial response, but this response was amplified by increasing temperature. We specifically demonstrate that (1) extracellular enzymatic activity and DOC consumption increased with warming, (2) this enhanced DOC consumption did not result in increased biomass production, since the increases in respiration were larger than for bacterial growth with warming, and (3) different DOC bioavailability affected the magnitude of the microbial community response to warming. We suggest that in coastal tropical waters, the magnitude of heterotrophic bacterial productivity and enzyme activity response to warming will depend partly on the DOC source bioavailability.

## Introduction

Heterotrophic bacteria are key players in many marine biogeochemical processes mainly due to their ability to degrade organic matter. Bacterial degradation of organic matter is largely controlled by its chemical composition and environmental factors such as temperature. An early study suggested a slower degradation of organic matter in polar compared to temperate and tropical marine environments (Pomeroy and Wiebe, 2001), with subsequent studies finding increasing microbial activity and/or organic matter degradation with temperature (e.g., López-Urrutia and Morán, 2007; Kirchman et al., 2009; Lønborg et al., 2016b; Morán et al., 2017 and references therein). Human activity is causing increases in global temperatures, with the ocean absorbing around 90% of this extra heat (Rhein et al., 2013). Climate models predict a mean increase in sea surface temperatures between 1 and 3°C by the end of this century (Rhein et al., 2013), with the warming rates of coastal zones exceeding by up to an order of magnitude those of the open ocean (Amos et al., 2003).

Tropical coastal waters are global hotspots of biogeochemical activity, receiving and processing approximately one order of magnitude more carbon, nitrogen, and phosphorus than temperate and polar regions (Brunskill, 2010), with the high activity being sustained by the relatively high river discharges, elevated temperatures and sunlight levels (Nittrouer et al., 1995). Despite the importance of tropical coastal waters, to date no study has investigated in detail how warming will impact biogeochemical processes in these low latitude systems. From a global warming perspective, tropical waters are special cases as they already experience elevated temperatures, which are increasing at a rate of about 70% of the average global ocean temperature (Lough, 2012). Whether tropical microbes will be impacted by increasing temperatures is still an unresolved question. Some studies suggest that they already operate close to their optima, hence further warming should not have any major impact on their metabolic rates (Wiebe and Pomeroy, 1999; Morán et al., 2017). However, other studies suggest that tropical marine microbes are expected to increase their activity under warming conditions (Mckinnon et al., 2017). Low latitude coastal waters are the ideal place to test these contrasting views, since presently they are characterized by the highest ocean surface temperatures on Earth.

Dissolved organic matter (DOM) is metabolically the most important source of energy (carbon) and nutrients (nitrogen and phosphorus among others) for heterotrophic bacterial growth (Lønborg et al., 2018a). The source of this DOM is thought to come partly from phytoplankton, dominated in tropical coastal waters by the picoplankton size-class (Furnas et al., 2005; Buitenhuis et al., 2012). However, in a subset of tropical systems, coastal vegetation is dominated by highly productive mangrove and seagrass ecosystems, which play key roles in the biogeochemical and material fluxes to the coastal zone (Kristensen et al., 2008; Alongi et al., 2013). How each of these DOM sources impact the degradation pathways is unknown, but a study in a temperate estuary has suggested that the source of organic matter could be important in regulating bacterioplankton carbon metabolism (Apple and Giorgio, 2007). Furthermore, Moran et al. (1991) demonstrated that the export of mangrove-derived DOM to coastal waters accounted for 10% of the total pool 1 km away from the source. Additionally, seagrass DOM exudation appears to sustain a large fraction of heterotrophic activity in coastal epipelagic waters (Ziegler and Benner, 1999). However, there are no studies determining the combined impact of warming and DOM substrates in tropical waters. But the fact that temperature and substrate availability act as interactive limiting factors for marine heterotrophic bacteria in large parts of the ocean (Pomeroy and Wiebe, 2001; López-Urrutia and Morán, 2007; Huete-Stauffer et al., 2015) suggests that focusing only on temperature might not suffice to accurately predict future changes. Therefore, establishing whether such links exist is critical to our understanding of the regulation of biogeochemical cycles in low latitude regions.

Our hypotheses in this study are that: (1) warming will increase the rates of microbial carbon processing in tropical coastal waters, but (2) substrate bioavailability will ultimately determine the extent of heterotrophic bacterioplankton activity increase (i.e., how much biomass will be produced by a 1°C temperature increase). To test these hypotheses, we conducted laboratory incubation experiments in which glucose, seagrass and mangrove DOM was added to surface seawater collected in the coastal region of the Great Barrier Reef, Australia (19°13′06′′S 147°8′21′′E). After inoculating the experiments with 1/10 of ambient bacterial assemblages, they were simultaneously incubated over 4 days at ambient temperature, and at 3°C below and above *in situ* values. Changes in heterotrophic bacterial abundance, activity (measurements of three extracellular enzymes, and of heterotrophic production via thymidine uptake) together with DOM bioavailability were measured to provide a comprehensive view of the temperature sensitivity of tropical planktonic bacteria and how these factors were impacted by the different DOM sources. Our results suggest that not only warming affected the metabolism of tropical heterotrophic bacterioplankton, but that the degree of the warming response changed dramatically depending on the DOM bioavailability.

## Materials and Methods

### Study Area

The Great Barrier Reef is situated on the continental shelf and slope of Australia’s North East coast, occupying a total area of 224,000 km^2^ (Hopley et al., 2007). Coral reefs and seagrasses occupy 7 and 20%, respectively of the shelf seabed, and adjoining mangrove forests cover a total area of 1044.8 km^2^ (Hopley et al., 2007). These estimates are derived from satellite images, but the value for seagrass meadows is imprecise owing to a lack of thorough validation. The open water body separating the reef area from the mainland is known as the Great Barrier Reef lagoon. This extensive lagoon has a water depth of around 10–20 m close to shore increasing to 40 m toward the reefs. The Great Barrier Reef region has a monsoonal climate, characterized by a wet summer (December–March) season and a dry winter season. The Great Barrier Reef shows considerable variation in surface water temperature (approx. range from 20 to 31°C), both seasonally and over its 15° of latitudinal extent. Despite the water column being generally well mixed, vertical temperature differences can occur in the deeper parts, with daily temperature changes exceeding 5°C during intrusion events from the Coral Sea (Andrews and Furnas, 1986). Mangroves and seagrasses are major contributors of material fluxes to the coastal zone of the Great Barrier Reef, thus both ecosystems play major roles in determining the biogeochemistry of the system, which might change in response to anthropogenic perturbations (Alongi et al., 2013). The system is furthermore characterized by rapid phytoplankton growth, protistan grazing and nutrient cycling (Furnas et al., 2005), with DOM representing around 80% of total carbon, nitrogen and phosphorus (Lønborg et al., 2018a).

### Experimental Setup

To test the impact of temperature and specific DOM sources on the Great Barrier Reef bacterial community, we added DOM from three different sources: glucose, DOM leached from the seagrass *Halodule uninervis* and from *Rhizophora stylosa* mangrove leaves. Marine surface water was used as a control. The seagrass and mangrove species were chosen as they are foundation species and are amongst the most common species in the Great Barrier Reef (Mckenzie et al., 2016).

The seawater sample was collected on October 18, 2016 (late dry season) using a 25 L acid cleaned Niskin bottle from 4 km off Cape Cleveland (19°13′06′′S 147°8′21′′E), Australia, a 20–30 m depth site away from direct human influence. The water was collected from 5 m depth and combined into two 50 L acid-washed containers. After collection, the seawater sample was kept in the dark until processed at the base laboratory. Water temperature was measured immediately after collection, while aliquots for the analysis of salinity were collected and measured in the laboratory using an Autosal 8400A. Water samples for chlorophyll *a* determination were collected by filtering seawater (200 mL) through a GF/F filter and analyzed after 90% acetone extraction with a Turner Designs 10000R fluorometer.

Seawater filtrations started within 1 h after collection. One part was gently vacuum-filtered (<10 KPa) in an acid-washed (2 mmol L^–1^ HCl and rinsed three times with Milli-Q water) glass filtration unit using a pre-combusted (450°C for 4 h) GF/C filters (nominal pore size 1.2 μm) to establish a starting community (total of 8 L was filtered) to be used in all experiments, which was kept in the dark close to *in situ* temperature (∼27°C) until used ∼2 h later. It is established that the use of filters with a similar pore size to GF/C filters may allow flagellates through (Cynar et al., 1985). In our experiment, we did not check for the presence of protists and can therefore not exclude they were present and impacted our results. The remaining seawater was gravity filtered through a dual-stage (0.8/0.2 μm) filter cartridge (Pall-Acropak Supor membrane), which had been pre-washed with Milli-Q water (>10 L). The seawater was hereafter used as the control experiment and to dilute the DOM obtained from a glucose solution, seagrass, and mangrove leaves.

The glucose (C_6_H_12_O_6_) stock solution was prepared in 0.2 μm-prefiltered MQ water. Fresh seagrass and mangrove leaves were collected in Cleveland Bay (19°13′05′′S 146°55′19′′E), Australia, brought back to the laboratory and rinsed thoroughly with 0.2 μm-prefiltered surface seawater. The seagrass and mangrove-derived DOM was thereafter extracted by adding ca. 25 g of dried leaves (60°C for 12 h in the dark) to a glass bottle containing 1 L of 0.2 μm-filtered seawater. After extraction (at 20°C for 24 h in the dark) the water was filtered through a pre–combusted GF/C filter and then through a dual–stage filter cartridge (0.8/0.2 μm, Pall-Acropak Supor membrane) to isolate the DOM fraction. The concentrations of dissolved organic carbon (DOC) and inorganic nutrient in all these solutions were then measured. The DOM sources were added to different 20 L carboys (glucose, seagrass, mangrove) to reach a DOC enrichment of ca. 40 μmol C L^–1^ (which corresponds to the approximate seasonal build-up of DOC in the Great Barrier Reef (Lønborg et al., 2017). These additions are obviously simplistic, as the natural DOC build-up consists of a mixture derived from a variety of sources, and not only from single species of mangrove and seagrass, or a single compound (e.g., glucose). On average, carbohydrates account for 6% (range 3–11%) of the DOC pool in the Great Barrier Reef, with DOC at inshore stations having concentrations between 70 and 126 μmol C L^–1^ (Lønborg et al., 2017). This results in an approximate carbohydrate concentration between 4 and 8 μmol C L^–1^, which is lower than our glucose addition. The GF/C filtered microbial culture was added in a ratio of 1 part of microbial culture, to 9 parts of water (control, glucose, seagrass, mangrove). The water was distributed into 180 different (45 per substrate) acid-rinsed glass bottles (500 mL) and incubated in the dark at three different temperatures (−3°C, *in situ*, +3°C), with 3 replicate bottles at each temperature and DOC treatment being collected for analysis at Days 0, 1, 2, 3, and 4. The incubations were sealed to eliminate the introduction of volatile organic compounds, but still had a ca. 100 mL headspace allowing air-water gas transfer. The bottles were not mixed during the incubation period but were mixed before each subsampling on Days 0, 1, 2, 3, and 4 to homogenize the sampled water. The incubations were performed in the dark at the National Sea Simulator (SeaSim) of the Australian Institute of Marine Science (AIMS), which is able to keep the temperature constant with an accuracy of ±0.1°C.

Unfiltered water from these bottles was used to follow changes in bacterial abundance (BA) and cell size, bacterial production (BP), and total extracellular enzymatic activity (EEA). Samples for the analysis of the dissolved phase were collected by filtration through prewashed (250 mL Milli-Q water) 0.2 μm filters (Pall, Supor membrane Disk Filter) to follow changes in dissolved inorganic nitrogen (DIN i.e., the sum of NH_4_^+^ and NO_3_^–^/NO_2_^–^), and phosphate (DIP: HPO_4_^–2^), and DOC. Water samples for DIN and DIP were collected in 20 mL acid washed polyethylene bottles and kept frozen (−20°C) until analysis. Sub-samples (10 mL) for DOC analysis were collected in pre-combusted (450°C, 12 h) glass ampoules and preserved by adding 50 μL of 25% H_2_PO_4_.

### Sample Measurements

DIN and DIP were determined using standard segmented flow analysis (Hansen and Koroleff, 1999). DOC concentrations were measured by high temperature combustion (720°C) using a Shimadzu TOC-L carbon analyzer. Prior to analysis, CO_2_ remaining in the acidified sample water was removed by sparging with O_2_ carrier gas. Three to five replicate injections of 150 μL were performed per sample. Concentrations were determined by subtracting a Milli-Q water blank and dividing by the slope of a daily standard curve made from potassium hydrogen phthalate and glycine. To avoid the small error associated with day-to-day instrument variability, all samples from each experiment (control, glucose, seagrass, mangrove) were analyzed on a single day. Using the deep ocean reference samples, we obtained an average DOC concentration of 43 ± 1 μmol C L^–1^. The average DOC concentration of deep ocean reference samples provided by the reference laboratory (Prof. Hansell Lab) are 41–44 μmol C L^–1^. The difference between the initial (DOC_0_) and minimum DOC (DOC_M__in_) concentration over the 4 days’ incubation was defined as the bioavailable DOC (BDOC).

In this manuscript, we use the term marine bacteria since archaea in coastal waters normally only contributed to a minor (generally <1%) part of the total marine prokaryote population (Delong, 1992). Samples for BA were fixed with 25% glutaraldehyde (0.5% final concentration) for 30 min at 4°C, flash frozen in liquid nitrogen and stored at −80°C until analyzed. Thawed samples were stained with the nucleic acid-specific dye SYBR Green I (Invitrogen-Molecular Probes) for 15 min in the dark and analyzed using a FACSVerse (BD Sciences) flow cytometer. The abundance was calculated after daily calibration of the flow cytometer’s flow rate as described in Gasol and Morán (2015). Bacterial cell volume (μm^3^) was obtained with an empirical calibration between cell diameter and mean right-angle light scatter in the flow cytometer assuming a spherical shape (Calvo-Díaz and Morán, 2006). Bacterial biomass (BB) was calculated by converting size into biomass using: pg C cell^–1^ = 0.12 × cell size^0.72^ (Norland, 1993).

BP was measured by [^3^H] thymidine incorporation (Fuhrman and Azam, 1980). A stock solution of [^3^H-methyl] thymidine (20 nmol L^–1^ final concentration) was added to three replicate 9.9 mL samples and 2 trichloroacetic acid killed samples. The samples were incubated in the dark at the respective temperature (−3°C, *in situ*, +3°C) for 1 h, 10 mL of ice-cold trichloroacetate (TCA) was thereafter added and samples were filtered onto 0.2 μm polycarbonate filters (pre-soaked in non-labeled thymidine), washed with 95% ethanol and autoclaved Milli-Q water. The filters were hereafter dried at room temperature (24 h) and mixed with 10 mL of scintillation fluid (Ultima Gold). The radioactivity incorporated into cells was counted using a Perkin Elmer Tri-Carb 2810 TR scintillation counter. The disintegrations per minute (DPM) of the TCA-killed blank were subtracted from the DPMs of the samples. The thymidine incorporated by bacteria was converted into carbon units using the theoretical conversion factor of 2 × 10^18^ cells mol^–1^ thymidine (Fuhrman and Azam, 1980) and actual BB estimates at each sampling day and experiment. The integrated BP values (BP_I__nt_) was calculated as the cumulative incorporation rate integrated over time. The cell specific BP (Cell BP) was calculated dividing the BP estimate by the cell abundance. In this calculation we were aware of the uncertainty associated with the presence of non-living cells, which could have caused an underestimation of the Cell BP.

The integrated bacterial growth efficiency (BGE in %) over the 4 days (BGE_Int_) was calculated as bacterial growth (BG, i.e., the net increase in BB between day 0 and the maximum, BG = BB_Max_ – BB_day__0_) divided by the BDOC:

(1) BGE=(BG/BDOC)×100

The hydrolysis of the fluorogenic substrate analogs L-leucine-7-amido-4-methylcoumarin (MCA),4-methylumbelliferyl (MUF)-β-D-glucoside and MUF-phosphate was analyzed to estimate potential activity rates of LAPase, BGase, and APase, respectively (Hoppe, 1983). LAPase is a proteolytic enzyme, BGase is a glycolytic enzyme, and APase is an esterase involved in the acquisition of P from DOM. The procedure was followed as previously described (Baltar et al., 2010, 2013). Briefly, EEA was determined after substrate addition and incubation using a spectrofluorometer with a microwell plate reader (Biotek Cytation 3 Imaging Multi-Mode Reader) at excitation and emission wavelengths of 365 and 445 nm, respectively. Samples (300 μL) were incubated in the dark at the respective temperature (−3°C, *in situ*, +3°C) for 1.5–3 h depending on activity. The increase in fluorescence over time was transformed into hydrolysis activity using a standard curve established with different concentrations of the fluorochromes MUF and MCA added to 0.2 μm filtered sample water. A final substrate concentration of 250 μmol L^–1^ was used, which was previously determined as saturating substrate concentrations. The integrated enzyme activity values (APase_Int_, BGase_Int_, and LAPase_Int_) were calculated as the cumulative incorporation rate integrated over time.

To calculate the activation energy (*E*_a_) in eV of BDOC and BG the obtained values were converted to rates by dividing the value by the day when the minimum (BDOC) or maximum values (BG) were obtained. For the BP and the activity of APase, BGase, and LAPase, the integrated rates were used to calculate the *E*_a_. We used integrated values to obtain an overall temperature impact and to ensure that the calculated temperature impacts could be compared with the BDOC and BG estimates which were derived from whole experimental values. Also, the calculation of the *E*_a_ is based on a relative change of rate activity with temperature, and therefore the overall change in values with temperature could be different (see Table 1) while the ratio between them remain constant. Temperature impacts were calculated using the Arrhenius law, which is defined by:

**TABLE 1:** Initial and final concentrations of dissolved inorganic phosphate (DIP) and nitrogen (DIN; sum of nitrate/nitrite + ammonium). The dissolved organic carbon (DOC) concentration at incubation day 0 (DOC_0_) and the minimum concentration (DOC_Min_) are also shown together with the bioavailable fraction (BDOC). Initial (BP_0_) and final (BP_4_) bacterial production (BP), and the 4 days integrated BP values (BP_Int_) are shown. The initial (BA_0_, BB_0_) and maximum (BA_Max_, BB_Max_) cell abundance (BA) and biomass (BB) are shown as well as the 4 days integrated growth in bacterial biomass (BG). The 4 days integrated enzymatic activities for alkaline phosphatase (APase_Int_), β-glucosidase (BGase_Int_) and leucine aminopeptidase (LAPase_Int_) are also shown. Values are averages of the three replicate bottles ± standard deviation.

	**Control**			**Glucose**			**Seagrass**			**Mangrove**		
**Temperature (°C)**	**−3°C**	**In situ**	**+3°C**	**−3°C**	**In situ**	**+3°C**	**−3°C**	**In situ**	**+3°C**	**−3°C**	**In situ**	**+3°C**
DIP_0_ (μmol P L^–1^)		0.08 ± 0.02			0.07 ± 0.02			0.03 ± 0.02			0.87 ± 0.01	
DIN_0_ (μmol N L^–1^)		0.26 ± 0.06			0.26 ± 0.03			0.24 ± 0.02			0.36 ± 0.02	
DOC_0_ (μmol C L^–1^)		83 ± 1			128 ± 2			128 ± 1			126 ± 1	
DOC_Min_ (μmol C L^–1^)	72 ± 1^∗∗^	69 ± 1^∗∗^	68 ± 2^∗∗^	118 ± 1^∗^	112 ± 4^∗^	105 ± 1^∗^	123 ± 1^∗^	121 ± 1^∗^	116 ± 1^∗^	102 ± 1^∗∗^	97 ± 1^∗∗^	92 ± 1^∗∗^
BDOC (μmol C L^–1^)	11 ± 1	14 ± 2	15 ± 2	9 ± 3	16 ± 6	23 ± 3	4 ± 1	8 ± 1	12 ± 2	24 ± 1	28 ± 1	33 ± 1
BP_0_ (μmol C L^–1^ d^–1^)	0.04 ± 0.01	0.05 ± 0.01	0.06 ± 0.01	0.02 ± 0.01	0.03 ± 0.01	0.05 ± 0.01	0.02 ± 0.01	0.03 ± 0.01	0.03 ± 0.01	0.01 ± 0.01	0.01 ± 0.01	0.01 ± 0.01
BP_4_ (μmol C L^–1^ d^–1^)	0.09 ± 0.01	0.12 ± 0.02	0.07 ± 0.01	0.09 ± 0.03	0.09 ± 0.01	0.11 ± 0.03	0.11 ± 0.01	0.12 ± 0.01	0.14 ± 0.01	1.78 ± 0.06	2.59 ± 0.18	4.08 ± 0.21
BP_Int_ (μmol C L^–1^)	0.42 ± 0.04	0.49 ± 0.04	0.50 ± 0.06	0.37 ± 0.04	0.41 ± 0.03	0.51 ± 0.06	0.38 ± 0.02	0.53 ± 0.03	0.57 ± 0.04	3.41 ± 0.20	5.08 ± 0.31	8.42 ± 0.93
BB_0_ (μmol C L^–1^)		0.09 ± 0.01			0.08 ± 0.01			0.10 ± 0.01			0.09 ± 0.01	
BB_Max_ (μmol C L^–1^)	0.36 ± 0.02^∗^	0.36 ± 0.02^∗^	0.36 ± 0.01^∗^	0.30 ± 0.03^∗^	0.37 ± 0.01^∗^	0.44 ± 0.03^∗^	0.58 ± 0.12^∗∗^	0.64 ± 0.11^∗^	0.63 ± 0.04^∗^	10.25 ± 1.08^∗^	9.42 ± 0.71^∗^	9.05 ± 0.92^∗^
BG (μmol C L^–1^)	0.27 ± 0.02	0.27 ± 0.03	0.27 ± 0.02	0.21 ± 0.04	0.28 ± 0.01	0.35 ± 0.04	0.48 ± 0.13	0.54 ± 0.12	0.53 ± 0.05	10.16 ± 1.09	9.33 ± 0.72	8.96 ± 0.93
APase_Int_ (nmol L^–1^)	12 ± 1	20 ± 1	30 ± 3	12 ± 1	24 ± 1	31 ± 2	14 ± 2	26 ± 3	30 ± 3	273 ± 22	341 ± 25	522 ± 40
BGase_Int_ (nmol L^–1^)	0.6 ± 0.3	1.2 ± 0.2	2.7 ± 0.5	2.0 ± 0.6	2.5 ± 0.6	3.4 ± 0.6	5.5 ± 1.7	6.8 ± 1.3	8.7 ± 1.9	102 ± 16	149 ± 7	185 ± 12
LAPase_Int_ (nmol L^–1^)	43 ± 3	41 ± 4	53 ± 5	66 ± 4	83 ± 8	88 ± 9	113 ± 21	201 ± 26	362 ± 23	241 ± 43	394 ± 31	1308 ± 130

*^∗^Value was obtained on day 3; ^∗∗^Value was obtained on day 4.*

(2) B=A×eT-Ea⁣/k×

where *B* is the metabolic rate; *A* is the theoretical metabolic rate if the rate was temperature independent; *E*_a_ is the activation energy; *k* is the Boltzmann’s constant (8.62 × 10^–5^ eV K^–1^); and *T* is the temperature in Kelvin. An estimate of *E*_a_ can be derived by the slope of a regression where the ln-transformed values were plotted against the inverse experimental temperature (1/*k* × T).

The relationship between temperature and biological rates has commonly been expressed with the factor by which a rate increases with a 10°C increase (*Q*_10_). In this study we assumed that the relationship between rates and temperature is exponential and we used the following function (Li and Dickie, 1987):

(3) $$Q_{10}=e^{\frac{E_{a}}{R}\,\frac{10}{T_{1}.T_{2}}}$$

where *T*_1_ and *T*_2_ are the temperatures at which the rates were measured.

Model I (ordinary least squares) linear regression analyses for calculating *E*_a_ values, analyses of covariance (ANCOVAs) for assessing significant *E*_a_ differences between experiments and paired *t*-tests were performed using JMP v11 software (StatSoft Inc.). As the *E*_a_ of the different metabolic rates were calculated with three values (i.e., the mean or integrated rates at 38.20, 38.58, and 38.97 1/KT values), we additionally tested whether the obtained *E*_a_ values using the three replicates (e.g., for BP or extracellular enzymatic activities) at a given time point (e.g., day 2) were comparable to the corresponding *E*_a_ values for the whole experimental period. A comparison between whole experiment *E*_a_ values and the mean values for the individual days 2 and 3 (where bacterial responses were fully developed) were strongly correlated (*r* = 0.89, *p* < 0.001, *n* = 16) and not significantly different (paired *t*-test, *p* = 0.44/*p* > 0.05, *n* = 16). This demonstrated that the *E*_a_ values calculated for the whole experiment and for individual days were comparable and that a consistent *E*_a_ was observed over the experimental period.

## Results

### Environmental Conditions and Dissolved Organic Carbon Degradation

The recorded surface seawater salinity (35.9), temperature (27.7°C), chlorophyll *a* (0.20 μg L^–1^), nutrient levels (DIP: 0.08 ± 0.02 μmol P L^–1^; DIN: 0.25 ± 0.06 μmol N L^–1^), and DOC concentrations (83 ± 1 μmol C L^–1^) were similar to the typical late dry season conditions in the Great Barrier Reef (Lønborg et al., 2016b).

**FIGURE 1:**
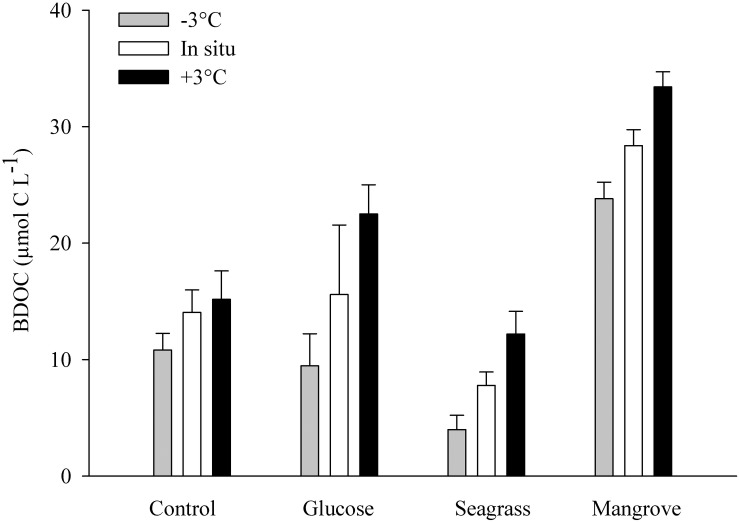
Average bioavailable dissolved organic carbon (BDOC; difference between initial and minimum DOC concentration) during the control, glucose, seagrass, and mangrove experiments, performed at –3°C (gray), *in situ* (white), and +3°C (black). Error bars represent standard deviations.

The DOC was added from different sources (glucose, seagrass, mangrove) to reach an enrichment of ca. 40 μmol C L^–1^, on top of the initial concentration found in the control, which was achieved in all experiments (Table 1). In all experiments the BDOC concentrations increased with warming (Figure 1 and Table 1). BDOC was highest in the mangrove experiment (range: 24–33 μmol C L^–1^), followed by the glucose (9–23 μmol C L^–1^), control (11–15 μmol C L^–1^), and seagrass (4–12 μmol C L^–1^) experiments (Figure 1 and Table 1). The calculated temperature sensitivity showed that in all experiments, except in the control (*p* = 0.17, *n* = 3), warming led to higher BDOC values, with the resulting activation energies varying between 0.44 ± 0.01 eV (Q_10_ = 1.8 ± 1.0) in the mangrove and 1.45 ± 0.15 eV (Q_10_ = 6.4 ± 0.8) in the seagrass experiment (Table 2). BDOC *E*_a_ values were significantly higher in the seagrass and glucose experiments relative to the other 2 DOC sources (seagrass = glucose > control = mangrove, ANCOVA, *p* = 0.003–0.013).

**TABLE 2:** The estimated activation energy (*E*_a_ in eV) and temperature coefficient, *Q*_10_, for bioavailable dissolved carbon (BDOC), bacterial growth (BG), productivity (BP_Int_), cell-specific bacterial production (Cell BP), alkaline phosphatase (APase_Int_), β-glucosidase (BGase_Int_), and leucine aminopeptidase (LAPase_Int_) enzymatic activity integrated over the 4 days incubation period in the different experiments (control, glucose, seagrass, and mangrove). Average values ± standard errors.

**Variable**		**Control**	**Glucose**	**Seagrass**	**Mangrove**
BDOC	*E*_a_ (eV)	0.44 ± 0.14	1.12 ± 0.09	1.45 ± 0.15	0.44 ± 0.01
	Q_10_	1.8 ± 0.8	4.2 ± 0.9	6.4 ± 0.8	1.8 ± 1.0
BG	*E*_a_ (eV)	0.01 ± 0.04	0.64 ± 0.05	0.14 ± 0.05	−0.16 ± 0.03
	Q_10_		2.3 ± 0.9	1.2 ± 0.9	
BP_Int_	*E*_a_ (eV)	0.23 ± 0.09	0.41 ± 0.09	0.53 ± 0.21	1.17 ± 0.09
	Q_10_	1.3 ± 0.9	1.7 ± 0.9	2.0 ± 0.8	4.5 ± 0.9
Cell BP	*E*_a_ (eV)	0.08 ± 0.10	0.22 ± 0.09	0.48 ± 0.05	1.07 ± 0.05
	Q_10_		1.3 ± 0.9	1.8 ± 0.9	3.9 ± 0.9
APase_Int_	*E*_a_ (eV)	1.25 ± 0.11	1.22 ± 0.29	0.97 ± 0.35	0.84 ± 0.16
	Q_10_	5.0 ± 0.9	4.8 ± 0.7	3.4 ± 0.6	2.9 ± 0.8
BGase_Int_	*E*_a_ (eV)	1.85 ± 0.12	0.72 ± 0.04	0.58 ± 0.02	0.77 ± 0.12
	Q_10_	10.7 ± 0.9	2.5 ± 0.9	2.1 ± 0.9	2.7 ± 0.9
LAPase_Int_	*E*_a_ (eV)	0.25 ± 0.23	0.38 ± 0.14	1.51 ± 0.02	2.20 ± 0.55
	Q_10_	1.4 ± 0.7	1.6 ± 0.9	6.9 ± 1.0	17.5 ± 0.5

### Bacterial Response to Sources of DOC and Warming

**FIGURE 2:**
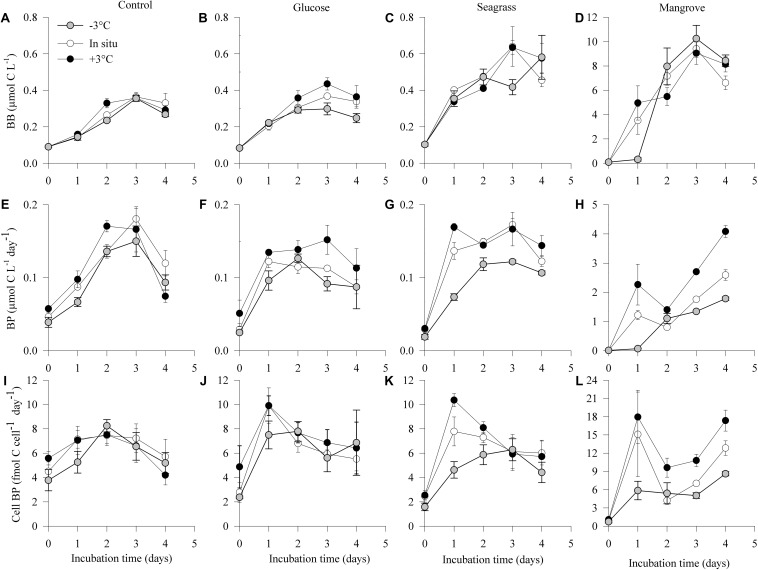
Dynamics of bacterial biomass (BB; **A–D**), production (BP; **E–H**), and cell-specific bacterial production (Cell BP; **I–L**) during the 4 days incubations in the different experiments (control, glucose, seagrass, and mangrove) performed at –3°C (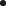), *in situ* (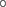), and +3°C (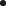). Values are mean values of three replicates and the Error bars represent standard deviations, where not visible error bars are within the symbol. Please note the difference in *Y*-axis scaling for the mangrove experiment.

As expected, initial bacterial biomass (BB_0_) did not differ between experiments (Figures 2A–D and Table 1), but it increased in all experiments following the consumption of DOC. The highest BB was reached in the mangrove experiment (day 3), followed by 16–25 times lower values in the seagrass, glucose, and control experiments (Figures 2A–D and Table 1). Warming only had measurable impacts on BG (see section “Materials and Methods,” Table 1) in the glucose experiments with an *E*_a_ of 0.64 ± 0.05 eV (Q_10_ = 2.3 ± 0.9), although there was also a minor response in the seagrass experiment but with a high error (0.14 ± 0.09 eV; Q_10_ = 1.2 ± 0.9, Table 2).

Initial bacterial production (BP_0_) was also similar in all experiments and temperatures but after 4 days (BP_4_) it was one order of magnitude higher in the mangrove experiment compared with the two other substrates and control (Table 1). In all experiments, BP showed a rapid initial increase to reach a rather stable level after 3 days of incubation with the exception of the mangrove experiment that kept increasing (Figures 2E–H). Warming increased the 4 days integrated BP (BP_I__nt_) rates in all experiments, with activation energies for BP_I__nt_ varying from 0.23 ± 0.09 eV (Q_10_ = 1.3 ± 0.9) in the control, followed by the glucose (0.41 ± 0.09 eV; Q_10_ = 1.7 ± 0.9) and seagrass (0.53 ± 0.21 eV; Q_10_ = 2.0 ± 0.8) experiments and up to 1.17 ± 0.09 eV (Q_10_ = 4.5 ± 0.9) in the mangrove experiment (Table 2). The ANCOVA analysis showed that the BP_I__nt_ activation energies were significantly higher in the mangrove relative to the other three DOC sources (mangrove > control = glucose = seagrass, ANCOVA, *p* = 0.007–0.027). The BP_I__nt_ and BG ratio was on average (±standard error) 1.1 ± 0.2, which is not significantly different from a 1:1 ratio, showing that both provide similar estimates of growth.

The Cell BP was largely driven by BP although changes in cell abundance modified slightly the pattern in the control and glucose experiments. Cell BP increased about 10 times in the mangrove experiment and about five times in the glucose and seagrass experiments (all at day 2), whereas the control only doubled by day 3 (Figures 2I–L). After days 2 and 3 the Cell BP decreased in all experiments except in the mangrove where it increased again on day 4. Warming impacted Cell BP in the glucose (0.22 ± 0.09 eV; Q_10_ = 1.3 ± 0.9), seagrass (0.48 ± 0.05 eV; Q_10_ = 1.8 ± 0.9), and mangrove (1.07 ± 0.05 eV; Q_10_ = 3.9 ± 0.9), experiments with the ANCOVA analysis demonstrating a significantly higher *E*_a_ values in the mangrove relative to the other two DOC sources and control (mangrove > control = glucose = seagrass, ANCOVA, *p* = 0.002–0.015).

**FIGURE 3:**
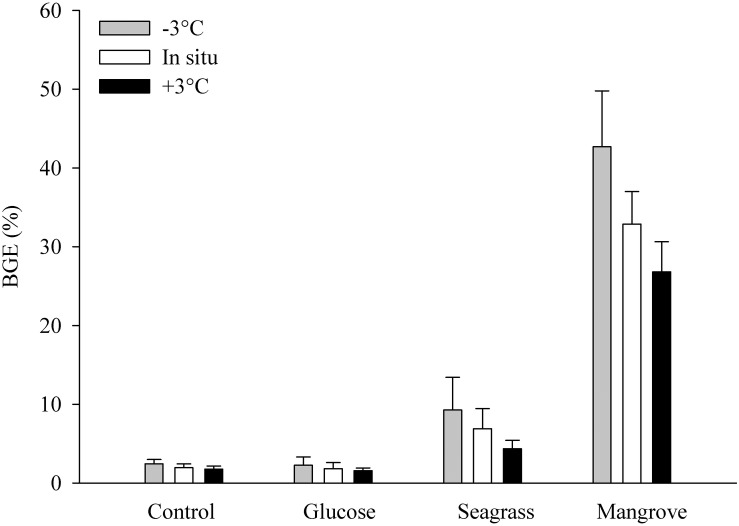
Bacterial growth efficiency (BGE in %) during the control, glucose, seagrass, and mangrove experiments performed at –3°C (gray), *in situ* (white), and +3°C (black). Values are mean values of three replicates and the error bars represent standard deviations.

Bacterial growth efficiency decreased with BDOC within the four experiments, resulting in highest values in the mangrove (between 27 ± 4 and 43 ± 7%) followed by the seagrass, and then by the glucose and control experiments with similarly low BGE values (∼2%; Figure 3). Warming consistently caused lower BGE within experiments (Figure 3), although the negative relationship between temperature and BGE was only significant for the seagrass experiment (*p* = 0.014, *n* = 3). Overall, BGE decreased between 0.12% (control and glucose) and 2.64% per°C warming (mangrove).

### Total Extracellular Enzymatic Activity

**FIGURE 4:**
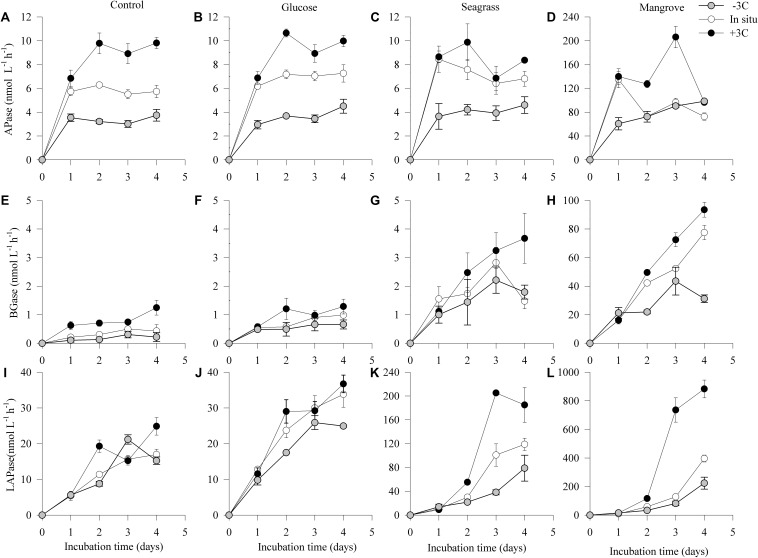
Dynamics of estimated potential enzyme activity rates of alkaline phosphatase (APase **A–D**), β-glucosidase (BGase; **E–H**), and leucine aminopeptidase (LAPase; **I–L**) during the 4 days incubations in the different experiments (control, glucose, seagrass, and mangrove) performed at –3°C (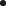), *in situ* (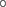), and +3°C (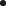). Values are mean values of three replicates and the Error bars represent standard error, where not visible error bars are within the symbol. Please note the difference in *Y*-axis scaling for all the enzyme activities in the mangrove experiment and the seagrass LAPase activity.

In general, the activity of LAPase (LAPase: 0–883 nmol L^–1^ h^–1^) was the highest followed by the activity of APase (APase: 0–206 nmol L^–1^ h^–1^) and BGase (BGase: 0–93 nmol L^–1^ h^–1^; Figure 4). All of the three EEAs showed an initial activity increase, particularly in the mangrove and seagrass experiments (Figure 4). APase activity increased in the mangrove relative to the control (by more than 20 times), but not in the other experiments (Figures 4A–D). BGase activity at the end of the experiment was approximately 4 and 90 times higher in seagrass and mangrove experiments relative to the control (Figures 4E–H). Similarly, LAPase activity at the end of the experiment was approximately 8 and 35 times higher in the seagrass and mangrove experiments compared to the control (Figures 4I–L). APase and BGase activities increased with warming in all experiments, except for APase at day 4 in the mangrove experiment, and for BGase in the seagrass experiment (Figures 4A–H). LAPase activity also increased in response to warming in the seagrass and mangrove experiments but was not clearly affected in the control and glucose experiments (Figures 4I–L). APase and BGase activation energies were highest in the control experiment (APase: 1.25 ± 0.11 eV; Q_10_ = 5.0 ± 0.9; BGase: 1.85 ± 0.12 eV; Q_10_ = 10.7 ± 0.9), and lowest in the mangrove (APase: 0.84 ± 0.16 eV; Q_10_ = 2.9 ± 0.8; BGase: 0.77 ± 0.12 eV; Q_10_ = 2.7 ± 0.9) and seagrass (APase: 0.97 ± 0.35 eV; Q_10_ = 3.4 ± 0.6; BGase: 0.58 ± 0.02 eV; Q_10_ = 2.1 ± 0.9) experiments, respectively. LAPase showed opposite trends to APase with highest *E*_a_ in the mangrove (2.20 ± 0.55 eV; Q_10_ = 17.5 ± 0.5) and lowest in the control experiment (0.25 ± 0.23 eV; Q_10_ = 1.4 ± 0.7) (Table 2). The ANCOVA analysis showed that the *E*_a_ was not significantly different between DOC sources for the APase (*p* = 0.20–0.87), while higher BGase *E*_a_ was found in the control compared to the three others (*p* = 0.003–0.006) and APase *E*_a_ was significantly higher in the mangrove and seagrass than in the other two sources (*p* = 0.008–0.049).

## Discussion

Determining the impact of temperature on heterotrophic bacterial degradation of DOC is essential to predict the response of tropical coastal waters to climate change (Ducklow et al., 2010; Lønborg et al., 2018b). After consumption, DOC has two possible pathways: it is either used for building up new BB (BP) or respired to CO_2_ (BR), with both processes being impacted by environmental conditions (e.g., temperature) and DOC chemical composition (López-Urrutia et al., 2006; López-Urrutia and Morán, 2007; Kirchman et al., 2009). Most previous studies have focused on how warming impacts BP or, less frequently, BR under natural environmental conditions, while other potentially important factors such as DOC quality or bioavailability have been neglected. Theoretically, with no other limitation, warming should result in higher enzymatic activity (Clarke and Fraser, 2004), followed by higher growth rates and ultimately abundances (Gillooly et al., 2001). However, in coastal marine systems bacterial DOC resources vary widely in their concentration and composition, depending on their ultimate source and age, and therefore the impact of warming may be far from homogeneous.

The application of the Arrhenius law has been broadened over the last decade under its reformulation within the metabolic theory of ecology (MTE; Brown et al., 2004). For example, the MTE has been previously used to calculate the *E*_a_ of ocean organic carbon degradation (Lønborg et al., 2018b), bacterial productivity (Lønborg et al., 2016a; Morán et al., 2017), bacterial specific growth rates (Huete-Stauffer et al., 2015) and the metabolic balance of the upper ocean (Brown et al., 2004; López-Urrutia et al., 2006; Lønborg et al., 2016a, 2018b). The MTE combines the effects of body size and temperature, suggesting that biogeochemical pathways depending on the process *E*_a_ might react differently to warming, possibly changing the overall flow of inorganic and organic material in marine ecosystems. According to this theory, the *E*_a_ of heterotrophic metabolism should approach a value of 0.65 eV (Brown et al., 2004; López-Urrutia and Morán, 2007). In our study the *E*_a_ values were generally higher or lower than the MTE predicted value. For lower values this can be interpreted in terms of e.g., inorganic and organic substrate stoichiometry and availability, so that the MTE predicted *E*_a_ are only met in substrate-sufficient conditions (Huete-Stauffer et al., 2015; Morán et al., 2018). Therefore, if the activity is limited due to e.g., a lack or an unfavorable balance between carbon and nutrients, an increase in temperature does not have an effect or a very limited one (low *E*_a_). That could be the interpretation for BDOC, BG and BP in the control experiment without extra DOC addition.

Extracellular enzymatic activities are the gatekeeper of the carbon cycle (Arnosti, 2011). However, the temperature dependence of marine extracellular enzymatic activity has not been studied in detail, and the few available estimates show highly variable *E*_a_ values (LAPase ca. −0.15–15.2 eV; BGase: 2.2–23.3 eV) (Vetter and Deming, 1994; Piontek et al., 2014). A recent global study has confirmed that generally, extracellular enzymatic activities increase with temperature, with higher *E*_a_ for BGase (0.12–0.39 eV) relative to LAPase (0.08−0.24 eV) (Ayo et al., 2016). In Baltar et al. (2017) we reported an increase in the total and cell-free LAPase in response to warming, which was ultimately affected by the bioavailability of DOC. However, in that study we did not calculate *E*_a_ values nor did we look at the effect of temperature on other EEA such as BGase or APase. In the present study, we calculated the *E*_a_ for all those enzymes, finding that ours lay within previously reported values. Most importantly, we also show that changing the DOM substrate did have some impacts on BGase while strongly increased LAPase (Figure 4 and Table 2). Of all the enzymes tested, APase showed the smallest range of *E*_a_ across all DOM substrates (0.94–1.25 eV), which is in accordance with previous findings (Ayo et al., 2016). When warming was the only perturbation of the community (control experiment), the enzymes had different *E*_a_ values. As the substrate that these enzymes target likely differ in their N and P content, it suggests that warming alone could affect the stoichiometry (i.e., the C:P and N:P ratios) of the degraded DOM macromolecules. However, when DOM from different sources were added, the *E*_a_ of the enzymes assessed changed relatively, which would cause further changes in the C:P and N:P ratios. For example, the higher *E*_a_ of LAPase relative to BGase would mean a shift toward higher degradation of proteins relative to carbohydrates, which in turn would decrease the C:N ratio of the degraded DOM pool. But it should be kept in mind that in our experiments the activity of only three classes of extracellular enzymes were tested, while in natural systems other enzymes would also be influenced by changing temperatures and BDOC levels, which combined would determine overall changes in the DOM stoichiometry.

The DOC bioavailability has previously been shown to vary depending on chemical composition and molecular size, and factors including inorganic nutrient availability, bacterial diversity (species and functions), mineral-particle associations, temperature and sun-light exposure (e.g., Amon and Benner, 1996; Thingstad et al., 1999; Del-Giorgio and Davies, 2003; Keil and Mayer, 2014). From our study it is clear that the BDOC levels had an overwhelmingly important effect on the measured response of the bacterial community, with the magnitude of this response being amplified by increasing temperature. It is noteworthy that the addition of seagrass extract reduced the BDOC even below the level of the control. By contrast, the mangrove leachate proved to be an excellent substrate with large amounts of DOC being consumed, stimulating BP as well as enzyme activities. In this study BDOC *E*_a_ varied largely, with values for the control and mangrove experiments (0.44 eV) being close to those found for DOC compounds degraded over days (∼0.39 eV; Lønborg et al., 2018b). In contrast, values for the seagrass and glucose (1.45 and 1.12 eV, respectively) experiments were closer to compounds degraded over longer timescales (∼1.21 eV; Lønborg et al., 2018b). The activation energies therefore varied with DOC substrates, which could be linked to differences in their chemical composition, with some compounds requiring more energy to be consumed (Brewer and Peltzer, 2017; Lønborg et al., 2018b). The high *E*_a_ for labile glucose was rather surprising. Higher *E*_a_ values have been related in soil studies to the presence of more refractory DOM (Davidson and Janssens, 2006). Since it is hard to imagine that glucose is more refractory than e.g., mangrove or seagrass DOM, we would rather argue that the bacterial community composition was actually different or that the processes involved in glucose degradation simply had a different, higher temperature sensitivity than the complex mixture of mangrove or seagrass DOM. In the Great Barrier Reef *Pelagibacter* spp., belonging to the SAR11-cluster of Alphaproteobacteria, represents between 20 and 84% of retrieved sequences (Alongi et al., 2015). Some *Pelagibacter* strains have been shown to lack glycolysis and therefore do not utilize glucose (Schwalbach et al., 2010). If these strains were present in relative high numbers in our experiments, they could have caused a lower BDOC values in the glucose experiment.

**FIGURE 5:**
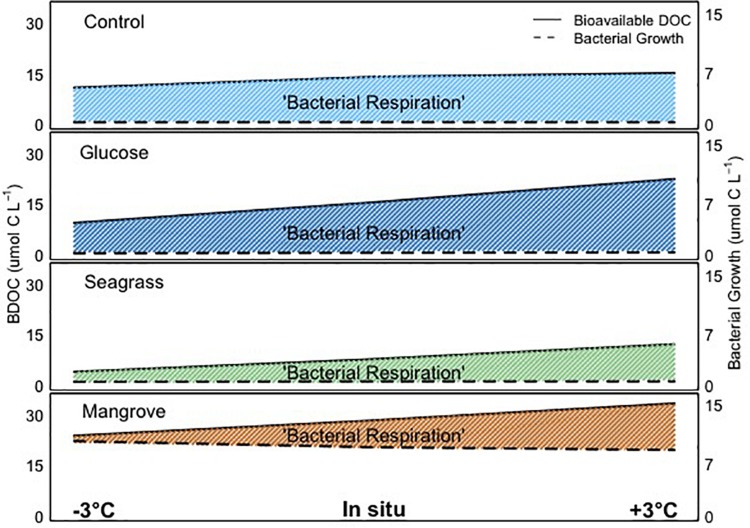
Graphical representation of the bioavailable dissolved organic carbon (BDOC; difference between initial and minimum DOC concentration), bacterial growth and bacterial respiration in all experiments (control, glucose, seagrass, and mangrove), over the range of temperatures (–3°C, *in situ*, and +3°C). The shaded areas highlight the bacterial respiration, as the difference between bioavailable DOC and bacterial growth. Please note that due to the scale the differences in bacterial growth with temperature are not visible.

The transfer of organic matter from DOC degradation to heterotrophic bacteria and subsequent, higher trophic levels ultimately depends on the BGE, which describes the relation between BP and BR. This ratio has previously been shown to vary greatly depending on environmental factors such as nutrient availability and trophic status (Rivkin and Legendre, 2001; López-Urrutia and Morán, 2007), DOM composition and quality (e.g., Goldman et al., 1987; Apple and Giorgio, 2007), previous sunlight exposure (Lønborg et al., 2016c) and temperature (Rivkin and Legendre, 2001; Lemée et al., 2002). For marine heterotrophic bacteria, the metabolic rates and consequently the energy demand theoretically grows with increasing temperature (e.g., Clarke and Fraser, 2004). Combined with substrate, temperature has been shown to limit bacterial activity (Pomeroy and Wiebe, 2001; Kirchman et al., 2009), with BGE suggested to be more strongly linked to resource (nutrients) availability than to temperature (López-Urrutia and Morán, 2007; Degerman et al., 2013). Overall, our ranges in BGE were comparable to values reported in other marine systems (Giorgio and Cole, 1998). However, with regard to the effect of substrate, it is clear from our experiments that the addition of mangrove DOC caused the largest increase in biomass and BGE (up to fivefold), while comparably low values were found in the seawater (control) and glucose experiments (Figure 3). Comparing all experiments, we found that higher BDOC led to lower BGE (within the four experiments), contrary to previous findings (e.g., Apple and Giorgio, 2007). This could be explained by differences in the DOC chemical composition, nutrient availability and/or the bacterial community composition. If we average the temperature dependences of BG and BP (i.e., the two independent methods we used for estimating the conversion of DOC into BB) shown in Table 2, the corresponding *E*_a_ values were different between them but were both lower than for BDOC in all experiments. This suggests that when carbon availability increased, it was used for catabolic (energy consumption) rather than anabolic (biomass production) processes. Thus, at higher temperature, tropical coastal bacteria were respiring the extra carbon away (Figure 5). Overall, we found an inverse relationship between BGE with BDOC and temperature within each experiment, demonstrating that increasing temperature decreased BGE and thereby hamper the transfer of carbon and energy up the food web. This apparent direct negative effect of temperature on BGE is contradictory to some studies (e.g., Vázquez-Domínguez et al., 2007) but in accordance with Rivkin and Legendre (2001), who reviewed literature values over a large set of environments differing in bacterioplankton community composition and trophic conditions. Since the initial bacterial community was the same in all experiments, although probably each DOM source selected for different dominant bacterial taxa, the consistent negative response of BGE to warming can be readily interpreted as a BR increase with temperature (Figure 5: because BDOC increased), while other factors, e.g., micronutrients or vitamins not measured in this study, could be limiting at higher temperatures, ultimately precluding an efficient transformation of consumed BDOC into microbial biomass.

In this study, comparing DOC amendments from different sources, we found for the first time in tropical waters that: (1) extracellular enzymatic activity and BDOC increased with warming, (2) this enhanced processing of DOC did not consistently result in higher BB production, since the increases in respiration were larger than for BG with warming in all experiments, and (3) different DOC source bioavailability influenced how the microbial community responded to warming (Figure 5).

Since heterotrophic bacteria play key roles in the marine carbon cycle, even minor changes in either their metabolism (i.e., caused by warming) or the availability of organic substrates (e.g., changes in the relative inputs of seagrass and mangrove-derived DOC assessed here) could have major impacts on future ocean biogeochemistry. Potential changes in primary producer stocks (Boyce et al., 2010) and community composition (Flombaum et al., 2013) due to climate change can strongly affect the proportion and composition of DOC released (Engel et al., 2011). Several modeling and experimental studies predict increases in the relative importance of dissolved primary production in direct response to warming (Engel et al., 2011; Huete-Stauffer et al., 2017). In coastal, shallow tropical waters, such as the Great Barrier Reef, seagrass, and mangrove ecosystems fuel part of the pelagic microbial activity (Alongi et al., 2013). However, as their relative coverage is estimated to decline by more than 40% within the next 100 years (Pendleton et al., 2012), the supply of two important carbon sources will be severely diminished. Our findings suggest that seagrass and mangrove extracts have contrasting bioavailability which consequently impacted the microbial community. Therefore, relative changes in extent and productivity of these systems by themselves would impact the biogeochemical cycles. But we also provide evidence that combined changes in substrate and temperature could indeed lead to less carbon being transferred up the food web, which would be instead lost to the atmosphere as CO_2_ due to the widespread decrease in BGE at elevated temperatures (0.1–2.6% per°C). In this context it should be borne in mind that our small-scale incubations can be useful to examine functional responses and causal relationships but should not be directly used to predict the manner in which warming will impact ecosystem scale processes, as many biological, physical and chemical interactions are not considered in these type of perturbation experiments. In the Great Barrier Reef short term physical processes, e.g., intrusion events from the Coral Sea can cause abrupt changes in temperature. It could therefore be argued that the microbial community is already well adapted to abrupt changes in temperatures as applied in these experiments. Ocean warming will also happen as a gradual heat absorption, while in our experiments we used an abrupt temperature shift of 3°C above or below the *in situ* temperature. Since microbes have short generation times and large population sizes, it is possible that a more gradual warming, as will be experienced with global warming, could allow a progressive acclimation or adaptation of the microbial community. In soils and lakes longer term heating experiments have demonstrated that microbial activity returns to pre-warming values within a few years, which has been linked with a depletion of labile organic matter and/or thermal adaptation (e.g., Bradford et al., 2008; Rousk et al., 2012). Acclimation or adaptation might also occur in the ocean, but studies testing the implications of such processes in the ocean are still lacking.

## Data Availability Statement

All datasets generated for this study are included in the article/supplementary material.

## Author Contributions

CL, FB, and XM contributed equally to the experimental work and sample analyses. All authors analyzed the data and wrote the manuscript.

## Conflict of Interest

The authors declare that the research was conducted in the absence of any commercial or financial relationships that could be construed as a potential conflict of interest.

## Abbreviations

APase: alkaline phosphatase
BA: bacterial abundance
BB: bacterial biomass
BDOC: bioavailable DOC
BG: bacterial growth
BGase: β-glucosidase
BGE: bacterial growth efficiency
BP: bacterial production
BR: bacterial respiration
Cell BP: cell specific bacterial production
DIN: dissolved inorganic nitrogen
DIP: dissolved inorganic phosphorus
DOC: dissolved organic carbon
DOM: dissolved organic matter
*E*_a_: activation energy
EEA: total extracellular enzymatic activity
LAPase: leucine aminopeptidase
MTE: metabolic theory of ecology
Q_10_: the factor by which a rate increases with a 10 ° C increase
